# Understanding the Interaction of Thermal, Rheological, and Mechanical Parameters Critical for the Processability of Polyvinyl Alcohol-Based Systems during Hot Melt Extrusion

**DOI:** 10.3390/pharmaceutics16040472

**Published:** 2024-03-28

**Authors:** Florian Hess, Thomas Kipping, Werner Weitschies, Julius Krause

**Affiliations:** 1 Merck Life Science KGaA, Frankfurter Straße 250, 64293 Darmstadt, Germany; 2 Department of Biopharmaceutic and Pharmaceutical Technology, Institute of Pharmacy, University of Greifswald, Felix-Hausdorff-Straße 3, 17487 Greifswald, Germany

**Keywords:** hot melt extrusion, polyvinyl alcohol, plasticizer, filaments, mechanical properties, viscosity

## Abstract

Hot melt extrusion (HME) is a common manufacturing process used in the pharmaceutical industry to improve the solubility of poorly soluble active pharmaceutical ingredients (API). The goal is to create an amorphous solid dispersion (ASD) where the amorphous form of the API is stabilized within a polymer matrix. Traditionally, the development of pharmaceutically approved polymers has focused on requirements such as thermal properties, solubility, drug–polymer interactions, and biocompatibility. The mechanical properties of the material have often been neglected in the design of new polymers. However, new downstream methods require more flexible polymers or suitable plasticizer polymer combinations. In this study, two grades of the polymer polyvinyl alcohol (PVA), which is already established for HME, are investigated in terms of their mechanical, rheological, and thermal properties. The mechanical properties of the extruded filaments were tested by the three-point bending test. The rheological behavior was analyzed by oscillating plate measurements. Thermal analysis was performed by differential scanning calorimetry (DSC). In addition, the solid and liquid plasticizers mannitol, sorbitol, triacetin, triethyl citrate, polyethylene glycol, and glycerol were evaluated for use with PVA and their impact on the polymer properties was elaborated. Finally, the effects of the plasticizers are compared to each other, and the correlations are analyzed statistically using principal component analysis (PCA). Thereby, a clear ranking of the plasticizer effects was established, and a deeper understanding of the polymer–plasticizer interactions was created.

## 1. Introduction

A large proportion of the active pharmaceutical ingredients (APIs) known today are poorly soluble in water, which poses challenges for the formulation of efficacious medicines [1]. Ting et al. [2] showed a breakdown of currently known APIs and pipeline substances in the Biopharmaceutical Classification System (BCS) [3]. A look at the distribution of APIs on the market, mentioned at the beginning, shows that 30% of the substances fall into BCS class II and 10% into BCS class IV. Looking at the current pipeline substances, i.e., the potential drug candidates, about 60–70% are BCS class II candidates, and an additional 10–20% are in BCS class IV [2]. The BCS class II candidates are of special interest. For them in particular, the solubility of the drug in biorelevant media is the decisive factor to act systemically and thus potentially be suitable as an oral form of administration [4,5].

To improve solubility, many methods are available [6]. The development of so-called amorphous solid dispersions (ASDs) is a particular focus in formulation development. In ASDs, the lattice structure of the API is opened up and the APIs are embedded in their amorphous state, for example, in a polymer matrix. This affects the drug solubility and dissolution rate, which can lead to an increased bioavailability [7,8,9,10]. For the production of amorphous solid dispersions (ASDs), various methods can be employed, such as spray drying [11], solvent evaporation [12], or high-energy milling [13]. Additionally, the supercritical fluid extraction technique has emerged as an alternative approach for the fabrication of ASDs, categorizing this method under adsorption on supports [14]. The other ASD production methods can be broadly classified into groups: solvent-based, fusion, or melting methods [15].

**Figure 1:**
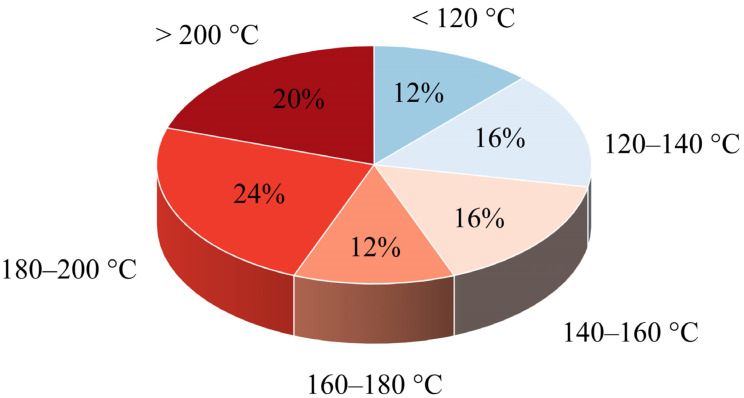
Melting point distribution of 24 APIs formulated as ASDs in currently marketed drug products (adapted from Alzahrani et al. [19]).

A well-established melt-based manufacturing process for ASDs is hot melt extrusion (HME) [16,17,18]. In this process, drug-excipient mixtures, often with polymers as a carrier matrix, are heated and mechanically mixed using one or more screws with special shear elements. The mixture becomes a viscous melt that is forced through a die. After cooling, the API remains dispersed in the polymer matrix, often in an amorphous state. This technique is already widely used in the pharmaceutical industry and several products are already on the market [19]. The APIs whose solubility could be improved by HME have different temperature-dependent process windows; these process windows must fit into the requirement profile of the polymer. The melting temperature (Tm) provides a preview of the temperature at which the APIs can be processed by HME. In Figure 1, the distribution of the melting points of the 24 APIs listed by Alzahrani et al. [19], which are processed via HME, is shown. Considering this broad distribution, it highlights how different temperature requirements must be met by suitable polymers in order to cover the variety of APIs.

In addition to the thermal properties, the development of pharmaceutically approved polymers has traditionally focused on requirements such as solubility, drug–polymer interactions, and biocompatibility. With the advent of new downstream methods such as strand pelletization, die-face pelletization, and film extrusion new mechanical demands were placed on the polymers [20]. This has been further emphasized with the emergence of 3D printing as a new technology in the pharmaceutical application field. Three-dimensional printing for pharmaceutical applications offers promising advantages, especially its high level of flexibility provides new approaches in the field of personalized medicine and in the acceleration of clinical trials. Therefore, there is increasing interest from pharmaceutical stakeholders to evaluate the different technologies and to develop products specifically designed for the different technologies [21]. Very prominent among the 3D printing technologies are the melt-based approaches, these include semisolid extrusion [22], direct powder extrusion [23], and fused deposition modeling (FDM) [24]. Melt-based 3D printing requires distinct polymer properties. Therefore, the focus lies in the identification of suitable polymers that have already been approved by medical regulatory authorities. For extrusion-based 3D printing systems, thermoplastics are extruded layer upon layer to create the desired three-dimensional object. Analogous to the HME process, the temperature processing range of the APIs is a decisive factor for these technologies. Especially for FDM printing, there is a special requirement. In this technology, beyond the thermal requirements, another bottleneck is often the brittleness of the polymers, as filaments are re-melted prior to the printing process. The tight path to the print head and the conventional feed mechanism made of gears require a suitable mechanical stability of the polymer filaments used. A lack of mechanical stability can be compensated with printer modifications [25] or plasticizer combinations [26,27].

Some pharmaceutical approved polymers were already investigated for these technologies including celluloses such as ethyl cellulose [28], hydroxypropyl cellulose [24], hypromellose acetate succinate [29], vinylpyrrolidone-vinyl acetate copolymers [30], polymethacrylates [31], and polyvinyl caprolactam derivatives [32]. A polymer that is frequently applied for 3D printing is polyvinyl alcohol (PVA) in pharmaceutical grades [33].

PVA is used in many pharmaceutical applications and is a well-characterized polymer for the HME [34,35,36] process. PVA is nontoxic, biocompatible, and water soluble. Often-used grades for hot melt extrusion are PVA 3-82 and PVA 4-88. In the nomenclature, the first number represents the viscosity in mPas of a 4% aqueous solution at 20 °C. This value is directly related to the molecular weight; thus, it is also an indicator for the viscosity of the melt. The second number stands for the percentage of hydrolyzed acetate groups and thus for the proportion of hydrophilic groups. Due to the difference in the hydrophilic/hydrophobic ratio, it is possible to partially interact with the API and prevent the API from precipitation [37]. 

In the literature, several solid and liquid plasticizers for PVA have already been described for different applications [38,39,40,41]. However, to the best of our knowledge, no research has comprehensively assessed the influence of plasticizers on the thermal, rheological, and mechanical properties of PVA in detail. To close this gap, we aimed to investigate and compare the two often-used PVA grades PVA 3-82 and PVA 4-88 regarding their key thermal parameters, their viscosity profiles in the melt, and the mechanical properties of the filaments produced via hot melt extrusion. Additionally, the influence of the plasticizers sorbitol (SOR), mannitol (MAN), glycerol (GLY), triacetin (TRI), triethyl citrate (TEC), and polyethylene glycol (PEG) in the grades PEG 300, PEG 400, PEG 600, and PEG 1000, and poloxamer (PLX) in grades 188 and 407 should be evaluated. The investigation of the plasticizers focuses in particular on the extension of the process window of the two PVA grades and furthermore, to compare the plasticizer influence on the mechanical properties of filaments. The different plasticizer combinations were ranked according to their influence on the rheological behavior of the melt and the mechanical properties of the PVA filaments. Finally, a statistical analysis by principal component analysis (PCA) should identify possible correlations between the data obtained and thereby provide a basic understanding of the interactions between plasticizer candidates and PVA. This is expected to provide new insights into the formulation development of PVA using HME.

## 2. Materials and Methods

### 2.1. Materials

Polyvinyl alcohol 3-82 (Parteck^®^ MXP 3-82) and Polyvinyl alcohol 4-88 (Parteck^®^ MXP 4-88) were supplied by Merck KGaA (Darmstadt, Germany). 

With the exception of poloxamer 407 (Kolliphor^®^P 407, BASF SE, Ludwigshafen, Germany), all other plasticizers used (poloxamer 188 (Parteck^®^ PLX 188), sorbitol (Parteck^®^ SI 150), mannitol (Parteck^®^ M200), PEG 300, PEG 400, PEG 600, PEG 1000, glycerol, triacetin, triethyl citrate) were supplied by Merck KGaA (Darmstadt, Germany).

### 2.2. Methods

#### 2.2.1. Blend Composition and Sample Preparation

For the binary systems containing 10 wt% liquid or solid plasticizer, the blends were ground manually in a mortar with a pestle. Each mixture was ground for 10 min with constant scraping of the material deposited on the edge of the mortar. This mixture is referred to below as the “physical mixture”. These mixtures were used in the DSC analysis and for the rheological investigations.

#### 2.2.2. Differential Scanning Calorimetry (DSC)

For the thermal analysis of the pure polymer and the physical mixtures, the DSC3+ from Mettler Toledo (Mettler Toledo GmbH, Gießen, Germany) was used. Approximately 5 mg of the sample was weighed into a 40 µL aluminum crucible. The crucible is perforated by the instrument before measurement to allow the escape of any gas that may be emitted. A second DSC crucible, perforated by hand, served as a reference crucible. A cyclical temperature program was run. Starting with a heating phase; for the solid plasticizers, a starting temperature of −20 °C was selected. The starting temperature of the mixtures with liquid plasticizers was −80 °C to identify strong Tg shifts and additional melting peaks. All samples with PVA 4-88 were heated up to 210 °C. The samples with PVA 3-82 were heated up to 200 °C. This was followed by a cooling phase down to the start temperature and then the second heating segment up to 250 °C. Between each heating and cooling element, the temperature was maintained for 2 min. The heating and cooling rate for all measurements was 10 K/min and a nitrogen purge gas flow of 50 mL/min was carried out. The data were analyzed with the STARe software (Version 16.00, Mettler-Toledo GmbH, Gießen, Germany).

#### 2.2.3. Melt Rheology

A Haake Mars 60 plate-plate rheometer from (Thermo Fisher Scientific Inc., Waltham, MA, USA) was used for rheological analysis of the pure polymers and the physical mixtures. It was equipped with aluminum single use plates with a diameter of 25 mm. Approximately 550 mg of the mixture was applied to the lower plate and then heated to 200 °C. The top plate was then dropped onto the sample to a gap height of 1 mm, any excess material that oozed out was trimmed off once the gap height was reached. The temperature of 200 °C was maintained for 5 min. The samples with PVA 4-88 were cooled to 170 °C and then reheated to 230 °C. The samples with PVA 3-82 were cooled to 150 °C and reheated to 230 °C. The viscosity, phase angle delta, loss modulus (G″), and storage modulus (G′) were determined in the range of 170 °C to 230 °C and from 150 °C to 230 °C, respectively. The heating rate was 5 °C/min for the initial cooling phase and 2 °C/min for the heating phase. The heating rate of the cooling phase was selected to be higher in order to keep the duration of the thermal stress lower. The upper plate moved, oscillating with a constant strain rate (0.1%) and frequency (6.28 rad/s) as determined in preliminary frequency sweep and amplitude sweep tests. Each test was performed three times. The data were analyzed with the HAAKE RheoWin software (Version 4.87.0006, Thermo Fisher Scientific Inc., Waltham, MA, USA).

#### 2.2.4. Hot Melt Extrusion of Filament Strands

To produce the filaments, solid (S) and liquid (L) plasticizers were differently handled. The mixtures with solid components were produced using a Turbula mixer (T2A, Willy A. Bachofen Maschinenfabrik, Muttenz, Switzerland). Mixing was performed at 46 rpm for 10 min. Mixing with the liquid components was performed directly in the extruder (Pharma 11, Thermo Fisher Scientific, Waltham, MA, USA). For this purpose, the liquid plasticizers were injected into the extruder barrel via a syringe pump (SP) (Harvard PHD-Ultra Syringe pump, Harvard Apparatus, Holliston, MA, USA). The pump was connected to a specially designed injector port built for injecting liquid located at the first connection port after the hopper. The density of the liquid was used to calculate the required flow volume to achieve a weight percentage of 10% plasticizer. The pure polymer was added through a gravimetric feeder (GF) (Congrav OP1-S, Brabender GmbH & Co. KG, Duisburg, Germany). The exact extruder parameters can be found in Table 1. 

The exact screw configuration for all experiments is shown in Supplementary Data Figure S1. The extruder nozzle was a custom-made extended nozzle with 20 mm length and 1.75 mm diameter. In all experiments, the extruder was operated at a screw speed of 200 rpm. The filament diameter was adjusted by the speed of the conveyor belt (Brabender GmbH & Co. KG, Duisburg, Germany) as closely as possible. To monitor the diameter, a 3-axis laser micrometer (Odac 33 Trio, Zumbach Electronic AG, Orpund, Switzerland) was integrated behind the conveyor belt. Strands with a diameter of 1.75 mm ± 0.05 mm were collected and used for further testing. 

#### 2.2.5. Mechanical Testing of the Filaments

To evaluate the influence of plasticizers on the mechanical properties of filaments, a 3-point bending test was carried out. The test was always performed on the same day as the extrusion with the Texture Analyser TA-XT from Stable Micro Systems (Stable Micro Systems Ltd., Godalming, UK). A literature method from Gottschalk et al. [25] was adapted to allow a direct assessment of filaments including the process history of the samples. For the test, the filaments were cut into 70 mm-long segments and then placed centrally on the test equipment. The contact surfaces were spaced 30 mm apart, and the impactor was positioned in the center. The impactor moved at a speed of 5 mm/s centrally between the contact surfaces onto the filament. When the edge of the impactor touched the specimen (release force: 0.049 N), the speed decreased from 5 mm/s to 0.1 mm/s. The tests were carried out ten times. The endpoint was defined by a breaking of filaments or the reaching of a maximum force. The data were analyzed with the Exponent software (Version 6.1.16.0, Stable Micro Systems Ltd., Godalming, UK). Flexural stress and flexural strain are calculated according to Prasad et al. [42] as follows:

$$\sigma_{f}=\frac{FL}{\pi R^{3}}$$

*σ_f_* = flexural stress [18];

*F* = applied force [N];

*L* = gap between contact surfaces [mm];

*R* = radius of the filament [mm].

$$\varepsilon_{f}=\frac{600sh}{L^{2}}$$

*ε_f_* = flexural strain [%];

*s* = deflection [mm];

*h* = diameter of the filament [mm];

*L* = gap between contact surfaces [mm].

#### 2.2.6. Principal Component Analysis (PCA)

PCA was conducted using the graphing and analysis software OriginPro 2022b (OriginLab Corporation, Northampton, MA, USA). No data were extrapolated, all data used were used from the analytical methods previously described. Since the data were obtained using different analytical methods, they were mean centered and scaled to unit variance in a correlation matrix. The eigenvalues of the principal components indicate which principal components cover the most information. 

## 3. Results 

### 3.1. Thermal Analysis

For the thermal study, the measured variables were the glass transition temperature (Tg), i.e., the temperature where the polymer transitions from a harder glassy state to a more viscous rubbery state, the melting point (Tm), the change in heat capacity (ΔCp) at the Tg, and the change in enthalpy (ΔH) at the melting point. The miscibility of the plasticizers with PVA was analyzed using the cyclic temperature program. For this purpose, a temperature program was run in which the melting point of all components was exceeded in the first heating cycle. Ideally, the substances would then combine to form a homogeneous mixture during cooling. Therefore, the focus herein is on the thermograms of the second heating cycle.

The results of the thermal analysis are shown in Table 2. The analysis of the pure PVA grades already showed differences in their thermal behavior. The Tg of PVA 3-82 was 62.6 °C and thus approximately 5 °C below that of PVA 4-88. A similar shift was seen in the melting point, which was 170.3 °C for PVA 3-82 and thus approximately 6 °C below that of PVA 4-88. 

Sorbitol and mannitol, as solid plasticizers, mixed well with both PVAs. In the thermograms of the second heating cycle, a single Tg and one melting point were observed. Mannitol and sorbitol strongly lowered the Tg of both PVA grades. Sorbitol had a slightly stronger effect on the Tg. The change in heat capacity at Tg was increased compared to that of neat PVA. The two polyols also had an influence on the melting point. Both lowered the melting point of PVA 3-82 slightly more than that of PVA 4-88. As with the Tg, sorbitol also showed a slightly stronger effect on the melting point. Greater differences could be seen in the effect on ΔH. Mannitol showed an increased ΔH with both PVA 4-88 and PVA 3-82. For PVA 4-88, sorbitol slightly increased ΔH, whereas for PVA 3-82, it decreased ΔH. 

The liquid plasticizers glycerol, triacetin, triethyl citrate, and PEG 300 also showed only one Tg and one melting point and thus homogeneous mixing with the PVA grades. As far as the Tg is concerned, the plasticizers showed different shifts among each other, but these shifts were quite similar in relation to the two PVA grades. Glycerol and PEG 300 showed the strongest Tg shift of about 24 °C for glycerol and about 18 °C for PEG 300. Most of the liquid plasticizers shifted the respective melting point of the two PVA grades to lower temperatures, the exception being the blends with PEG 300. PEG 300 had a minor effect, increasing the melting point of the PVA blend 4-88 by about 2 °C and that of the PVA blend 3-82 by 4 °C. Triacetin showed the greatest effect on PVA 3-82, lowering its melting point by about 40 °C. With PVA 4-88, triethyl citrate showed the greatest effect, lowering the melting point by about 43 °C. The liquid plasticizers all reduced ΔH at the melting point but increased the change in the ΔCp at Tg (Table 2).

**Figure 2:**
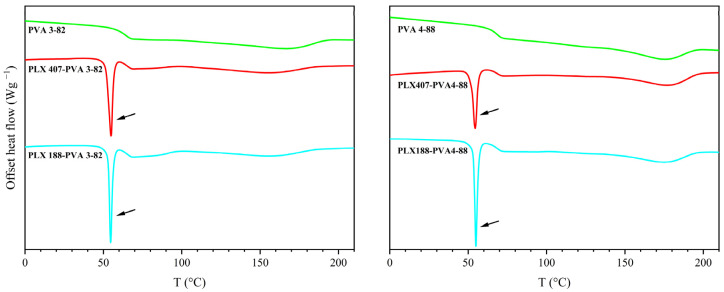
Thermogram of the second heating cycle with PVA 3-82 and PVA 4-88 and 10 wt% poloxamer 407/188 (PLX 407/PLX 188).

The two types of poloxamer, poloxamer 188 and poloxamer 407, could not be combined with PVA; each exhibited an additional melting point in combination with both PVAs, as can be seen in Figure 2 (indicated by the black arrows).

**Figure 3:**
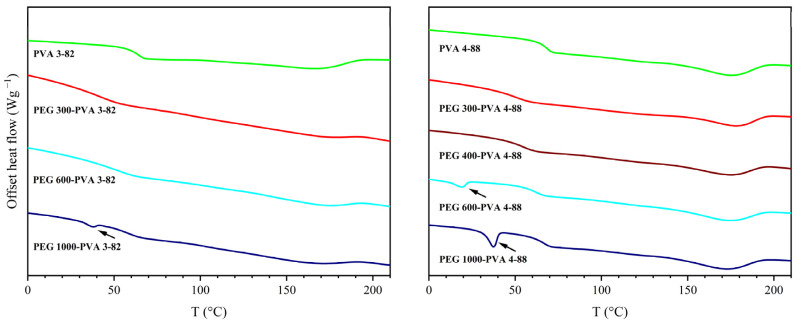
Thermogram of the second heating cycle with PVA 4-88 and PVA 3-82 and different polyethylene glycol (PEG) grades with a ratio of 10 wt% PEG.

The thermal investigation of the PEG grades is shown in Figure 3. Differences between the different PEG grades, as well as between their interaction with the two PVA grades were detected. For PVA 3-82, only PEG grades below PEG 1000 exhibited a single melting point. From PEG 1000 onwards, two melting points appeared in the thermogram. With PVA 4-88, this limit shifted, with two melting points appearing from PEG 600 onwards.

The exact values for the poloxamer and PEG blends can be found in Table 3. However, it should be noted that in the poloxamer measurements the first melting point was very close to the Tg, partly overlapped, and therefore, ΔCp could only be approximately determined. In addition to the poloxamer peaks in the thermogram, there was also no influence on Tg of the PVA grades.

**Figure 4:**
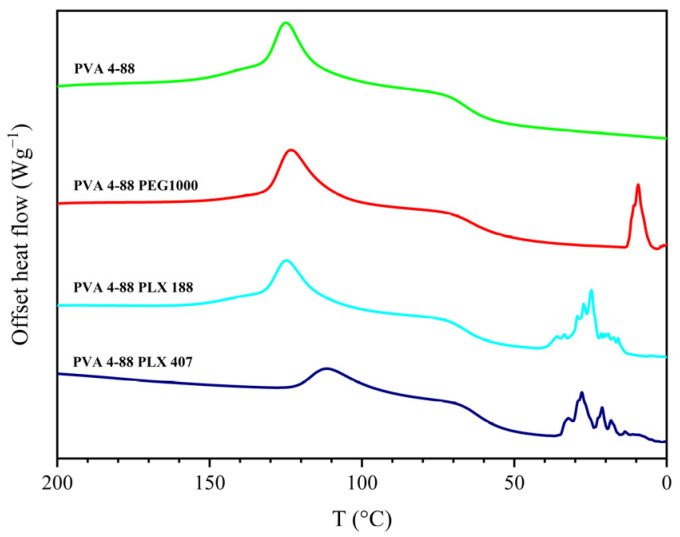
Exemplary thermogram of the cooling segment with blends that could not be mixed compared to the neat PVA 4-88.

It was also observed that the binary systems that displayed two melting peaks showed a characteristic jagged region in the cooling cycle. This is illustrated in Figure 4 for the two poloxamers and PEG 1000 in combination with PVA 4-88.

Based on their demonstrated miscibility by DSC, glycerol, triacetin, triethyl citrate, PEG 300, mannitol, and sorbitol were selected as candidate plasticizers for further experiments.

### 3.2. Melt Rheology

**Figure 5:**
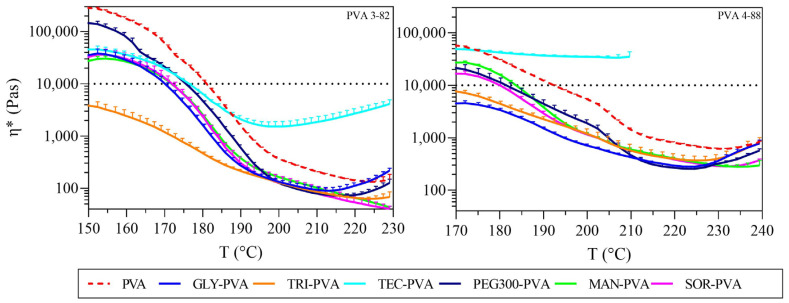
Complex viscosity against temperature, left: PVA 3-82 and PVA 3-82/plasticizer systems; right: PVA 4-88 and PVA 4-88/plasticizer systems, the PVA–plasticizer systems are denoted using abbreviations corresponding to their respective plasticizers: glycerol (GLY), triacetin (TRI), triethyl citrate (TEC) polyethylene glycol (PEG), mannitol (MAN), and sorbitol (SOR), (means of n = 3, + SD), plasticizer proportion is 10 wt%.

In hot melt extrusion of polymers, a melt viscosity threshold of 10,000 Pas is often considered the upper process limit to maintain torque within an appropriate range [43]. Since viscosity decreases with increasing temperature for most polymers, the process window in terms of processing temperature for extrusion can be estimated by measuring viscosity over a specified temperature rise. A comparison of the polymers revealed different processing ranges for the two PVA grades (Figure 5). PVA 3-82 entered the processable range of lower than 10,000 Pas beginning at lower temperatures (starting at 181 °C), and PVA 4-88 required higher temperatures to enter the processable range (starting at 192 °C). Accordingly, different process windows applied to the two PVA grades. This indicates that PVA 3-82’s absolute viscosity generally decreased more with increasing temperature compared to PVA 4-88.

Considering the solid plasticizers in the individual PVA types, both mannitol and sorbitol shifted the processability limit downward by about 10 °C for each PVA grade.

In the case of liquid plasticizers, subtle differences were observed in their effect on the viscosity of the PVA grades. Triacetin had the greatest effect on PVA 3-82, lowering the start of its processability to below 150 °C. The formulations containing glycerol and PEG 300 showed a reduction in melt viscosity. In contrast, the blends containing triethyl citrate showed an unusual behavior compared to the other plasticizers. Initially, viscosity decreased; however, beyond 190 °C, it increased, surpassing the viscosity of neat PVA 3-82.

When comparing the melt viscosities of the liquid plasticizer with PVA 4-88, the greatest shift was observed in the glycerol/triacetin blends. Here, the lower extrusion temperature limit shifted by more than 25 °C. PEG 300 showed a decrease in viscosity with a temperature drop of almost 10 °C. Triethyl citrate exhibited distinct properties, significantly increasing melt viscosity. Additionally, decomposition with bubble formation was observed starting at 205 °C. The graph was plotted only up to this point, because the appearance of bubbles made accurate measurement impossible.

**Figure 6:**
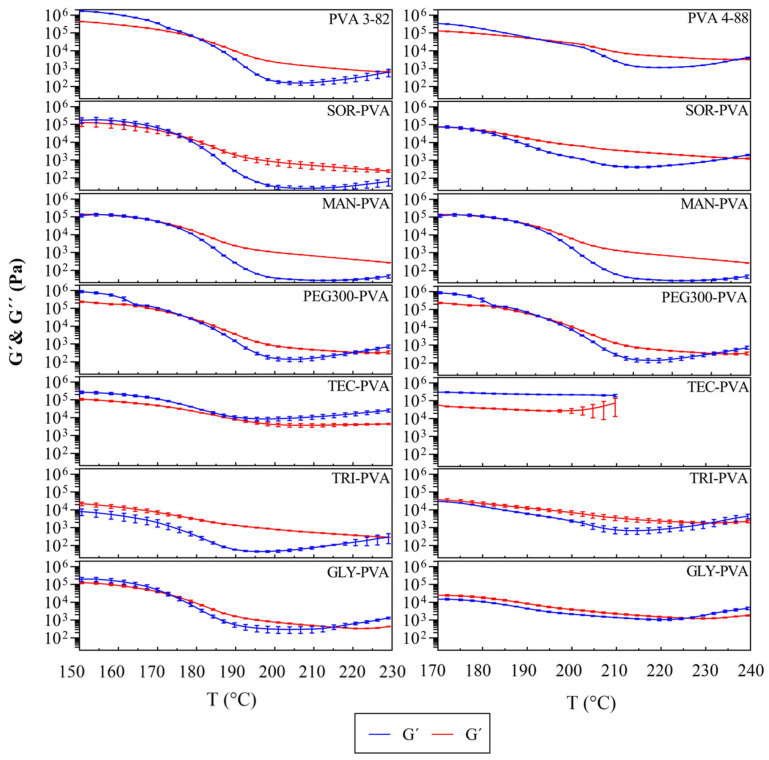
Storage modulus (G′) and loss modulus (G″) of PVA 3-82 and PVA 3-82/plasticizer systems against temperature, the PVA–plasticizer systems are denoted using abbreviations corresponding to their respective plasticizers: glycerol (GLY), triacetin (TRI), triethyl citrate (TEC) polyethylene glycol (PEG), mannitol (MAN), and sorbitol (SOR), means of n = 3, plasticizer proportion is 10 wt%.

Figure 6 illustrates the relationship between the storage modulus (G′) and the loss modulus (G″). The storage modulus (G′) is a measurement of the energy stored and recovered [44], representing the elastic part of the material response to shear. The loss modulus (G″) is a measurement of the energy dissipated or lost as heat [37], representing the viscous part of the material response to shear. At the crossover points, there is a balance between G′ and G″, indicating a structural change in the viscoelastic behavior of the polymer melt.

For PVA 3-82, the system transitioned to a viscoelastic liquid above 180 °C, and for PVA 4-88, above 192 °C. Mannitol in combination with PVA 3-82 resulted in a crossover at about 169 °C. The crossovers of the PVA 4-88 and the mannitol blends occurred at about 183 °C. The combinations with triacetin and glycerol in combination with PVA 3-82 exhibited a viscoelastic range over the entire measurement range, starting at 150 °C. The same applied for the blends with PVA 4-88, but here a shift to a predominantly elastic behavior was observed at 226 °C for the glycerol mixture, and at 232 °C for the triacetin mixture. The blends with PEG 300 started their viscoelastic range nearly at the same temperatures as the pure PVA grades but exhibited an earlier second crossover. Triethyl citrate did not show optimal extrusion conditions, since, for both PVA grades, an elastic system was present over the entire temperature range. Due to degradation signs, the graph only extends up to 210 °C. All values can be found in Table 4. 

**Figure 7:**
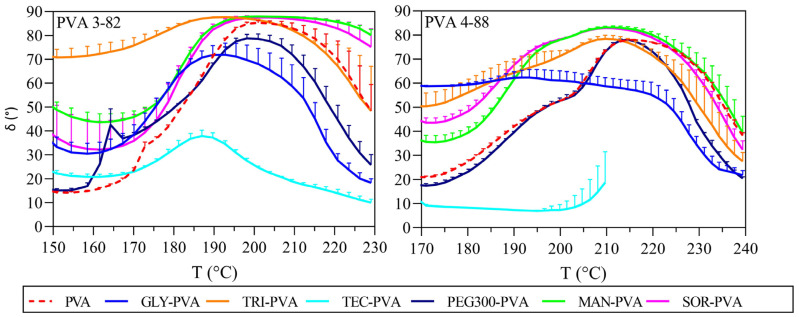
Phase-angle delta against temperature, left: PVA 3-82 and PVA 3-82/plasticizer systems; right: PVA 4-88 and PVA 4-88/plasticizer systems, The PVA–plasticizer systems are denoted using abbreviations corresponding to their respective plasticizers: glycerol (GLY), triacetin (TRI), triethyl citrate (TEC) polyethylene glycol (PEG), mannitol (MAN), and sorbitol (SOR) (means of n = 3, + SD), plasticizer proportion is 10 wt%.

The phase angle δ describes the behavior between the vector of the complex shear modulus G*, the x-axis that represents G′, and the y-axis is formed by G″. The phase angle δ can range from 0° to 90°. An angle of 0° would represent an ideal elastic behavior with no viscous portion. An angle of 90° would represent an ideal viscous behavior with no elastic portion. The term viscoelastic solids is used when G′ is greater than G″ [45]. This corresponds to a range at the phase angle of 0° to 45°. Viscoelastic liquids are defined as having a G′ smaller than G″, which corresponds to a phase angle of 45° to 90°. The phase angle δ clearly displays the influence of the plasticizers on the PVA and is shown for both PVA grades in Figure 7. Considering the phase angle progression of mannitol and sorbitol in comparison with neat PVA 3-82, the 45° limit for viscoelastic liquids was reached about 10 °C earlier compared to pure PVA 3-82. Additionally, the decrease in phase angle was delayed, resulting in a noticeable plateau. When considering the phase angle, the mixture with triacetin stands out. Triacetin showed a high phase angle over the whole period of the measurement. Triacetin changed the PVA 3-82 in such a way that the viscoelastic properties desired for melt extrusion were already achieved from at least 150 °C. The phase angle curves of the PVA 4-88 blends showed in general a slightly lower phase angle δ compared to that of PVA 3-82 and their binary systems. The blends with the polyols did not show a delayed drop, but the ideal viscoelastic range was observed to be reached at lower temperatures. Triacetin and glycerol shifted the viscoelastic range most strongly to lower temperatures, but the phase angle then decreased earlier at 220 °C. The viscoelasticity of pure PVA 4-88 was stable at this temperature. 

### 3.3. Hot Melt Extrusion

**Figure 8:**
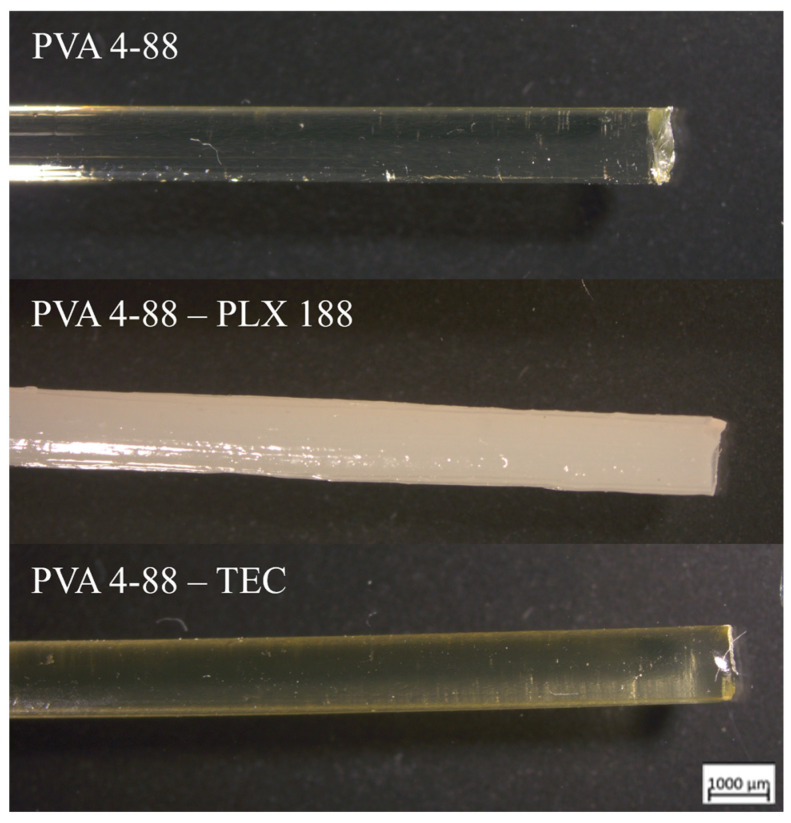
Microscopic images of the filaments. Exemplified by pure PVA 4-88 and the binary systems PVA 4-88-poloxamer 188 (PLX 188) and PVA 4-88-triethyl citrate (TEC).

Both PVA grades were extrudable without the need for further additives. Transparent homogeneous filaments with a diameter of 1.75 mm ± 0.05 mm could be produced. The filaments had a smooth surface, and their color was transparent with a very pale-yellow tinge. In terms of external appearance, no differences were found between PVA 3-82 and PVA 4-88. Figure 8 shows an example of a microscopic image of a PVA 4-88 filament, both with and without plasticizer.

The plasticizers glycerol, triacetin, triethyl citrate, PEG 300, mannitol, and sorbitol could also be extruded in combination with both PVA grades. The plasticizers appeared to have no influence on the filaments’ optical appearance, except for triethyl citrate. Triethyl citrate showed a stronger yellow color in combination with PVA 4-88. This effect was not observed with PVA 3-82.

The plasticizers, which were identified as immiscible with the PVA grades by the previous DSC measurements, also exhibited a two-phase character in the filaments. Despite the shear energy input from the extruder’s mixing elements, the blends could not be formed into homogeneous filaments. The blends with Poloxamer 407 and Poloxamer 188, as well as with PEG 1000, showed white opaque filaments whose diameter varied strongly, making it impossible to produce uniformly diametric filaments suitable for three-point bending tests. 

### 3.4. Mechanical Testing of the Filaments

**Figure 9:**
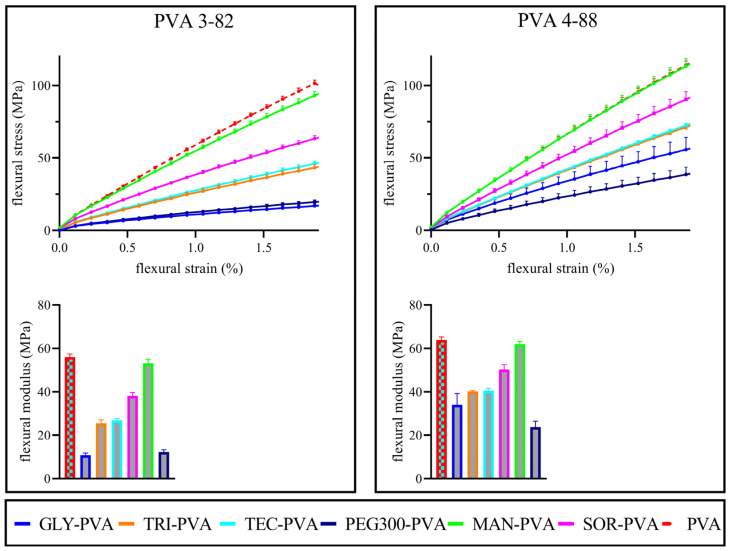
Above: Flexural stress–strain diagram of the respective PVA grades and the PVA–plasticizer systems, mean ± SD, n = 10; Below: Flexural modulus of the respective PVA and the PVA–plasticizer systems, The PVA–plasticizer systems are denoted using abbreviations corresponding to their respective plasticizers: glycerol (GLY), triacetin (TRI), triethyl citrate (TEC) polyethylene glycol (PEG), mannitol (MAN), and sorbitol (SOR), mean ± SD, n = 10, plasticizer proportion is 10 wt%.

The flexibility of the filaments was assessed via flexural stress–strain curves. The flexural modulus, calculated from the slope between 0.25% and 0.5% flexural strain, provided measurable numerical values. The flexural modulus approximates to the E-modulus (Young’s modulus) [46]. The lower the flexural modulus, the less rigid the sample. Both PVA grades showed similar basic flexibility in the stress–strain diagrams, as can be seen in Figure 9 and Table 5 and their flexural modulus. The solid plasticizer candidates mannitol and sorbitol differed in their influence on the flexibility of the filaments. Mannitol with PVA 4-88 showed no increase in flexibility compared to the pure polymer. In combination with PVA 3-82, it also showed only a very slight increase in flexibility. Although sorbitol has a high structural similarity to mannitol, it showed a clear difference compared to the mannitol blends in its effect on the flexibility of the filaments, i.e., sorbitol increased the flexibility. Among the liquid plasticizers, triacetin and triethyl citrate had the least plasticizing effect. Their stress–strain curves were very close to each other. Glycerol and PEG 300 showed the highest increase in flexibility. In combination with PVA 3-82, PEG 300 showed the strongest effect. In combination with PVA 4-88, glycerol showed a stronger effect. Details can be found in Table 5.

**Figure 10:**
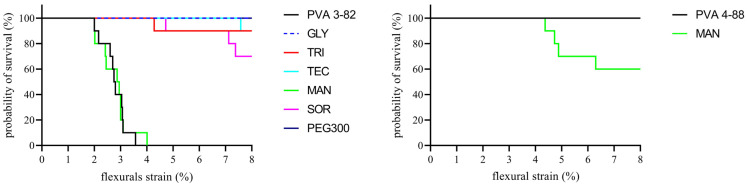
Kaplan–Meier plot to estimate the mechanical integrity of PVA and the PVA/plasticizer filaments, the PVA–plasticizer systems are denoted using abbreviations corresponding to their respective plasticizers: glycerol (GLY), triacetin (TRI), triethyl citrate (TEC) polyethylene glycol (PEG), mannitol (MAN), and sorbitol (SOR), n = 10, plasticizer proportion is 10 wt%.

While both PVAs showed similar flexibility, there were significant differences in the brittleness of the polymers. This is illustrated by a variation in the Kaplan–Meier plot, as can be seen in Figure 10. While no strand of the PVA 4-88 filaments broke during the entire test, 50% of the PVA 3-82 filaments broke after only 3% strain and all strands broke after 3.5% strain. The influence of plasticizers on the mechanical properties showed differences between the PVA grades. The mannitol formulation exhibited brittleness very similar to that of pure PVA 3-82. Sorbitol showed some improvement in flexibility, but 30% of the filaments broke. Following these, triacetin and triethyl citrate formulations each resulted in a 10% breakage loss during the test. PEG 300 and glycerol were the best-performing formulations, reducing the brittleness the most with no breakage during the test. The evaluation of brittleness was more difficult with PVA 4-88, since no filament broke even with the neat polymer (Figure 9). The plasticizer systems showed the same behavior. No filament broke in the three-point bending test. An exception were the filaments with the combination of mannitol and PVA 4-88; here, an embrittlement of the polymer could be observed. Thus, the first breaks already occurred at a deflection of the filament by approximately 4.5% flexural strain. Across the entire measurement range, 30% of these filaments broke.

### 3.5. PCA

For the PCA, the correlations of the following measured data among each other were investigated: From the thermal analysis, the glass transition point and the melting point, as well as the enthalpy change at these temperatures, were considered (Table 3). From the rheological analysis, the values of the viscosity, storage modulus, loss modulus, and phase angle at the temperature of the melt during extrusion were used. For PVA 3-82 blends, it was 180 °C; for PVA, 4-88 190 °C. From the three-point-bending tests, the values for flexural stress and flexural modulus and flexural strain at maximum flexural stress were used.

**Figure 11:**
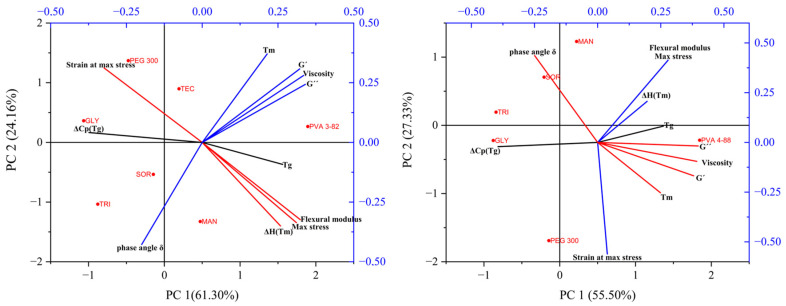
PCA biplot (PC1 vs. PC2) (**left**): for PVA 3-82 and PVA 3-82/plasticizers systems, (**right**): for PVA 4-88 and PVA 4-88/plasticizers systems, the PVA–plasticizer systems are denoted using abbreviations corresponding to their respective plasticizers: glycerol (GLY), triacetin (TRI), triethyl citrate (TEC) polyethylene glycol (PEG), mannitol (MAN), and sorbitol (SOR).

The PCA with the PVA 3-82 (Figure 11) showed two principal components together representing 87.5% of the information. The most significant principal components are displayed by the shoulder points in the Scree diagram (Supplementary Data Figure S2). Plotting the two main components against each other shows the correlations within one measurement method. Thus, the values of the mechanical examination flexural modulus and the maximum stress are close to each other in the lower right quadrant, while the strain at which the stress is maximum is located in the upper left quadrant. This indicates a negative correlation. This can be explained by the fact that with brittle material, the filament breaks earlier. Therefore, the stiffer filaments have high force values and low strain values at the same time. A similar distribution can be seen in the values for the rheological measurements. The viscosity, storage modulus (G′), and loss modulus (G″) together lie in the upper right quadrant. Negatively correlated with this is the relationship between G″ and G′, and the phase angle δ. This measurement, indicative of the melt’s viscous or elastic behavior, appears inversely related to viscosity. The plasticizers, which lower the viscosity the most, show a higher phase angle at the processing temperature. They exhibited more viscous behavior, whereas systems with lesser impact on viscosity showed predominantly elastic behavior. The results of the thermal analysis are not directly adjacent. They are distributed over three quadrants. Looking at the first principal component, we see a correlation between the viscosity, storage modulus (G′), loss modulus (G″), and the mechanical results of flexural modulus and maximum stress. The thermal parameters Tg and Tm and the enthalpy change at the melting point are also in an unspecific correlation with the values. Also considering the second principal component, representing 24.16% of the information, a clear clustering of the viscosity parameters with the melting point obtained from the DSC analysis and a second clustering of the mechanical properties and the enthalpy at the melting point can be seen. Accordingly, the influence of a plasticizer on the melting point, in addition to the Tg shift [47], is decisive for its viscosity-lowering properties. There seem to be different mechanisms for the correlation between mechanical and thermal properties. The plasticizers with the greatest influence on filament flexibility and brittleness, PEG 300 and glycerol, both showed a strong reduction in Tg (Table 3). However, it also appears that the enthalpy and thus the base semi-crystallinity of the PVA was a correlated parameter with the mechanical properties. The red points mark the plasticizer systems and the pure PVA on the coordinates of the determined variables of the main components. No clear clustering of the plasticizers could be determined, but the points give a good visual overview of their influences on the mechanical, thermal, and rheological properties. The lack of specific clusters indicates very different influences of the plasticizers but may also be due to the quantity of the used combinations. Statistical evaluations usually benefit from larger datasets.

For the trials with PVA 4-88, the PCA (Figure 11) comprises three principal components that together represent 93.59% of the information: PC1 41.34%, PC2 35.85%, and PC3 16.40%. Most of the information can be obtained from the first two principal components. Basically, similar observations can be seen as for PVA 3-82. However, the strain is no longer completely opposite at maximum stress. This can be explained by the fact that with PVA 4-88, the strands did not break during the test, except for the mannitol combinations, thus somewhat distorting the test for brittleness. The remaining points are positioned similarly to those for PVA 3-82 but with a slightly larger deviation. The extracted eigenvectors for both PCAs can be found in the supplementary data.

## 4. Discussion

### 4.1. Thermal Analysis

The thermal events determined in this work are in line with published results of DSC measurements performed under similar conditions with the two PVA grades [48,49].

In addition to the classical measurands Tg and Tm, the enthalpy change at the melting point, which is represented by the area under the curve (AUC) of the endothermic melting peak, provides further information about the thermal characteristics of the polymers to be investigated. It is already described in the literature that the enthalpy is directly related to the crystalline structures within the polymer melts. An increased AUC at the melting point stands for increased crystallinity [50,51]. The two PVA types differ in their thermal properties. The Tg, Tm, and ΔH of PVA 4-88 are higher than those of PVA 3-82. An explanation could be provided by the combination of different approaches. Thermal studies with polyethylene glycol (PEG) of different molecular weights also conclude that the thermal parameters Tm and ΔH are directly influenced by the molecular weight and thus the chain length of the PEGs. Here, a higher chain length refers to a higher Tm and ΔH [52]. PVA should be considered in a more differentiated way; indeed, the first number of the nomenclature indirectly stands for a different molar mass. However, the degree of hydrolysis has a higher impact on Tm and ΔH than the molecular weight of the PVA. Previous studies have shown that an increased degree of hydrolysis leads to an increased Tg, Tm, and ΔH [53]. This phenomenon is most likely due to the higher intramolecular hydrogen bonding forces resulting from the increased number of hydroxyl groups.

The different degrees of hydrolysis can also provide an explanation for the different mixing behavior with the longer-chain PEG chains. The limit at which PEG could be mixed with the respective PVA was PEG 600 for PVA 3-82 and PEG 400 for PVA 4-88. In the case of PEG, the number in the nomenclature stands for the molecular weight. This suggests that the size of the polymer chains is decisive for the miscibility with the two examined PVA grades in the melt. One reason why slightly longer chains can be mixed with PVA 3-82 could be the lower degree of hydrolysis. The presence of approximately 6% more acetate groups appears to stabilize longer PEG chains. 

The influence of the molecular weight of the plasticizers on their miscibility with polyvinyl alcohol (PVA) is further confirmed by the results with the two poloxamers. A poloxamer is a co-polymer composed of polyethylene oxide and polypropylene oxide units. PLX 188 has an approximate molar mass of 8400 g/mol, while PLX 407’s is about 14,600 g/mol. Their molar masses are significantly higher than the molar masses of PEG 400 or PEG 600 and could not be mixed with either of the two PVA grades. This hypothesis is also supported by the jagged cooling curve which indicates that the included PLX and PEG regions are crystallized independently of the PVA [54].

### 4.2. Rheological Analysis

To be able to determine the appropriate temperature ranges for extrusion, rheological investigations are one of the key tests for HME [55,56,57]. The two PVA grades already offer a broad processing range suitable for APIs that melt at very high temperatures. To extend this process window and to be able to cover more lower-melting active ingredients with the PVA types used, glycerol and triacetin had the greatest impact. Since the viscosity of a liquid or a polymer melt is a function of the intermolecular forces that restrict molecular movement, the observed decrease in viscosity indicates that the intermolecular resistance to flow has been reduced by the disentanglement of long-chain molecules [58]. The PCA analysis leads to the conclusion that this happens most due to the lowering of the Tm by the plasticizer. This effect is strongest for PVA 3-82 in combination with triacetin and in the case of PVA 4-88 in combination with glycerol. Based on the assumption that the process range for PVA 3-82 extends below 150 °C with triacetin, approximately 28% of the APIs currently processed with HME might also be compatibly processed using PVA. 

The storage modulus (G′), loss modulus (G″), and the phase angle δ are additional representations of the extrudability of a polymer, as they reflect not only the thermodynamic aspect of the material but also its kinetic attributes [55]. Changes in the curve of the phase angles are indicative of a thermal event; therefore, it can be assumed that the drop in the phase angle after reaching the maximum indicates another change in the viscoelastic behavior which is possibly linked to the beginning of decomposition. Considering the phase angle progression of PVA 3-82 in the presence of mannitol and sorbitol in comparison with neat PVA 3-82, we see that the drop was delayed, and there was a regular plateauing of the phase angle. This could indicate that mannitol and sorbitol have a temperature stabilizing effect on PVA 3-82. When considering the phase angle, the mixture with triacetin stands out. Triacetin showed a high phase angle over the whole period of the measurement. Triacetin changed the viscoelasticity of PVA 3-82 in a way that the desired properties for melt extrusion were already achieved from at least 150 °C. The phase angle curves of the PVA 4-88 blends showed that PVA 4-88 does not decompose up to an approximate temperature of 235 °C. It seems that the blends with the polyols did not show a further temperature stabilizing effect for PVA 4-88. Triacetin and glycerol shifted the viscoelastic range most strongly to lower temperatures, but the level then drops earlier at 220 °C, which could indicate an earlier degradation temperature of these systems. An earlier drop in the combination with PEG 300 suggests that this plasticizer also reduces the temperature stability of PVA 4-88. The viscoelasticity of pure PVA 4-88 was stable at this temperature. Outside the viscoelastic region, the predominantly elastic behavior could lead to an insufficient mixing due to a lack of deformation of the polymer during extrusion.

### 4.3. Hot Melt Extrusion

The process temperatures were adjusted individually for the two PVA grades. The temperature settings for the binary plasticizer systems remained constant for each PVA grade in order to generate filaments that were processed with comparable process parameters. The binary systems used could be extruded with the specified process parameters for torque and pressure at the die. Previous HME studies have investigated PVA grades across a wide range of hydrolysis degrees, from 33% to 88% [36]. This work focuses on the PVA grades with the higher degrees of hydrolysis (82% and 88%) as these are reported to perform better in the production of ASDs [36]. 

The plasticizer concentration was kept constant at 10 wt% in order to be able to rank the different plasticizers against each other. The literature reports that increasing the proportion of liquid plasticizers like glycerol enhances the flexibility of PVA 4-88 [59]. However, the increase in plasticizer content is often accompanied by an even greater decrease in Tg. This can lead to physical instability of ASDs. In future studies, it will be necessary to find the balance between the necessary plasticizing effect and the stability of the ASD.

### 4.4. Mechanical Testing of Filaments

The effects of glycerol on PVA films have already been investigated in the work of Fu et al. [60]. This work concludes that the plasticizing effect of glycerol on PVA films is due to the replacement of hydrogen bonds between polymer chains with those between polymer chains and glycerol. The greater the number of hydrogen bonds with the plasticizers, the weaker the bonds between the polymer chains. This also leads to a decrease in Tg with an increase in plasticizer content. A similar observation was also made with plasticizers in combination with chitosan [61]. These observations are in line with some of the observations made in this work: the two strongest plasticizers in terms of mechanical strength of the filaments were PEG 300 and glycerol, both of which lowered the Tg (Table 2) and thus the intramolecular hydrogen bonds between the polymer chains the most. Mannitol and sorbitol are stereoisomers that differ only in the position of one of their six hydroxy groups, but they differ strongly in their effect on the mechanical properties of PVA filaments. 

Mannitol lowers the Tg by a large amount but leads to increased brittleness in combination with PVA 4-88, and equal brittleness with PVA 3-82. Sorbitol, on the other hand, shows a plasticizing effect on both PVA grades. Similar differences between mannitol and sorbitol were also observed in combination with starch [62]. An increased stiffness of the starch films was observed by the addition of mannitol. Furthermore, sorbitol was found to form more hydrogen bonds to the starch molecules than mannitol. According to their work, this may be due to sorbitol’s molecular structure and hydroxyl group conformation being more similar to glucose units compared to mannitol. Therefore, sorbitol could be more inclined to form more hydrogen bonds with starch. This cannot be transferred one-to-one to the interactions with PVA; here, another special characteristic of the two polyols could provide a reason for the different effects. It is known that in aqueous solution mannitol adopts a planar zig-zag conformation, whereas sorbitol adopts a bent-chain conformation [63]. This is one approach to explain the different solubilities of mannitol and sorbitol in water. This difference could also explain the varied plasticizing effects of the two polyols on PVA. Accordingly, the more bent conformation of sorbitol could replace more intermolecular hydrogen bonds between the PVA chains than the planar zig-zag configuration of mannitol.

It seems that there could be other effects influencing the brittleness of the PVA filaments. The statistical analysis of the parameters showed evidence of other mechanisms, albeit with much less influence on the stiffness of the filaments, such as an increased correlation between the partial crystallinity of the PVA grades and the flexibility of the polymer filaments. In particular, the plasticizers triacetin and triethyl citrate showed a strong reduction in crystallinity, but they had less influence on the Tg. On the PVA filaments, they had a plasticizing effect.

## 5. Conclusions

This study investigated the thermal, rheological, and mechanical properties of two PVA grades, PVA 4-88 and PVA 3-82. PVA 3-82 is a rather new polymer grade with an adapted degree of hydrolysis and thus far has been less documented in the literature. As expected, PVA 3-82 demonstrated a lower melt viscosity compared to PVA 4-88, enabling extrusions to be performed at lower temperatures. The most notable differences between the two PVA grades were observed in the mechanical stability of the extruded materials. PVA 3-82 extrudates showed a lower mechanical strength and exhibited a higher brittleness compared to PVA 4-88. These properties are usually required for downstream steps via milling or grinding. Other downstream processes require more flexible intermediates. 

Furthermore, this study identified suitable plasticizers for each PVA grade and evaluated their use in melt extrusion. The plasticizers exhibited varying effects on the melt viscosity and the mechanical properties of the extrudates. Consequently, formulations for different application areas can be optimized using various polymer–plasticizer combinations. Triacetin showed the greatest viscosity-reducing effect on PVA 3-82, while both glycerol and triacetin exhibited the most prominent effect in combination with PVA 4-88. These plasticizer combinations substantially expanded the temperature-related processing window of PVA, potentially offering advantages in formulations with temperature-sensitive APIs. The mechanical properties of the PVA extrudates for both grades were most influenced by the addition of glycerol or PEG 300. These combinations not only reduced the stiffness of the extrudates but also decreased the brittleness of PVA 3-82 the most. Thus, these plasticizer combinations can optimize the PVA grades for various downstream methods with high mechanical requirements.

Statistical analysis using PCA revealed correlations between the thermal, rheological, and mechanical properties. The shift in the melting point strongly correlated with the melt viscosity of the PVA–plasticizer systems. The plasticizer combinations that had the most significant effect on the TG of the PVA grades showed the greatest impact on the mechanical stiffness. Additionally, the influence of plasticizers on the semi-crystallinity of PVA emerged as a less prominent but still notable factor affecting the mechanical properties. These insights can be advantageous for formulators to individually optimize their formulations regarding their respective downstream technology.

## Figures and Tables

**Table 1:** Extrusion parameters and temperatures for hot melt extrusion (S: solid plasticizer; L: liquid plasticizer).

Formulation	Feed Rate (GF) [kg/h]	Feed Rate (SP) [kg/h]	Zone 1 [°C]	Zone 2 [°C]	Zone 3 [°C]	Zone 4 [°C]	Zone 5 [°C]	Zone 6 [°C]	Zone 7 [°C]	Die [°C]
PVA 4-88 (S)	0.2	-	80	150	200	200	200	200	200	210
PVA 4-88 (L)	0.18	0.02	80	150	200	200	200	200	200	210
PVA 3-82 (S)	0.2	-	80	150	190	190	190	190	190	190
PVA 3-82 (L)	0.18	0.02	80	150	190	190	190	190	190	190

**Table 2:** Results from the second heating cycle of the DSC analysis (means of n = 3, ±SD).

Plasticizer	PVA 3-82				PVA 4-88			
	Tg [°C]	ΔCp [K/Jg^−1^]	Tm [°C]	ΔH [Jg^−1^]	Tg [°C]	ΔCp [K/Jg^−1^]	Tm [°C]	ΔH [Jg^−1^]
none	62.6 (±0.3)	0.659 (±0.099)	170.3 (±23.0)	5.450 (±0.453)	67.3 (±0.4)	0.628 (±0.038)	176.7 (±0.4)	11.620 (±0.511)
GLY	38.2 (±2.6)	0.938 (±0.106)	150.3 (±3.2)	3.550 (±0.427)	44.3 (±1.3)	0.774 (±0.034)	166.2 (±1.1)	10.067 (±0.891)
TRI	56.3 (±1.2)	0.912 (±0.023)	129.4 (±1.6)	3.153 (±0.244)	61.9 (±0.9)	0.791 (±0.015)	148.5 (±2.8)	5.4770 (±0.468)
TEC	61.5 (±0.6)	0.871 (±0.036)	155.1 (±0.5)	2.953 (±0.080)	70.7 (±0.4)	0.762 (±0.012)	133.4 (±0.4)	6.250 (±0.646)
PEG 300	44.0 (±0.5)	0.896 (±0.041)	174.0 (±2.5)	2.917 (±0.759)	48.8 (±1.0)	0.725 (±0.045)	178.6 (±0.4)	9.973 (±0.264)
MAN	52.1 (±0.1)	0.801 (±0.013)	157.1 (±0.3)	5.983 (±0.278)	53.3 (±0.5)	0.695 (±0.019)	168.4 (±0.2)	13.603 (±0.146)
SOR	49.9 (±0.1)	0.895 (±0.024)	153.9 (±0.5)	4.745 (±0.106)	50.3 (±0.5)	0.728 (±0.036)	166.9 (±0.7)	12.730 (±0.495)

**Table 3:** Results from the second heating cycle of the DSC analysis (means of n = 3, ±SD).

Plasticizer	PVA 3-82						PVA 4-88					
	Tg [°C]	ΔCp [K/Jg^−1^]	Tm_(1)_ [°C]	ΔH_(1)_ [Jg^−1^]	Tm_(2)_ [°C]	ΔH_(2)_ [Jg^−1^]	Tg [°C]	ΔCp [K/Jg^−1^]	Tm_(1)_ [°C]	ΔH_(1)_ [Jg^−1^]	Tm_(2)_ [°C]	ΔH_(2)_ [Jg^−1^]
PLX 188	66.2 (±1.5)	0.375 (±0.024)	54.6 (±0.0)	12.27 (±0.215)	158.3 (±1.0)	4.37 (±0.424)	68.0 (±0.0)	0.414 (±0.014)	55.0 (±0.1)	13.06 (±0.953)	177.3 (±0.0)	8.65 (±0.236)
PLX 407	65.0 (±0.2)	0.393 (±0.022)	54.8 (±0.2)	11.82 (±1.764)	158.78 (±1.5)	4.35 (±0.367)	68.0 (±0.3)	0.386 (±0.023)	54.6 (±0.1)	11.34 (±0.520)	178.68 (±0.0)	8.79 (±0.191)
PEG 400	N/A	N/A	N/A	N/A	N/A	N/A	53.9 (±0.3)	0.617 (±0.020)	N/A	N/A	176.5 (±1.0)	8.40 (±0.446)
PEG 600	51.9 (±0.6)	0.813 (±0.069)	N/A	N/A	173.2 (±2.7)	3.43 (±0.719)	60.9 (±0.3)	0.488 (±0.031)	19.3 (±0.1)	1.13 (±0.225)	175.6 (±0.6)	9.11 (±0.284)
PEG 1000	55.7 (±1.7)	0.490 (±0.058)	37.7 (±0.3)	0.65 (±0.093)	169.0 (±2.2)	3.87 (±0.823)	65.5 (±0.5)	0.557 (±0.071)	37.3 (±0.0)	6.26 (±1.927)	173.5 (±0.4)	10.3 (±0.277)

**Table 4:** Crossover points of storage modulus (G′) and loss modulus (G″), means of n = 3.

Plasticizer	PVA 3-82		PVA 4-88	
	1. Crossover [°C]	2. Crossover [°C]	1. Crossover [°C]	2. Crossover [°C]
none	180.1	228.3	192.3	237.1
GLY	N/A	N/A	N/A	225.9
TRI	N/A	226.4	173.6	231.9
TEC	N/A	N/A	N/A	N/A
PEG 300	176.5	220.6	192.7	229.5
MAN	169.0	N/A	183.4	237.8
SOR	174.4	N/A	177.6	235.7

**Table 5:** Flexural modulus, maximum stress, and the strain at maximum stress (means of n = 10).

Plasticizer	PVA 3-82			PVA 4-88		
	Flexural Modulus [18]	Max. Stress [18]	Strain at Max Stress [%]	Flexural Modulus [18]	Max. Stress [18]	Strain at Max Stress [%]
none	59.0	137.8	2.701	64.8	196.0	5.693
GLY	11.1	29.8	6.396	34.0	95.3	5.464
TRI	25.3	74.5	5.500	39.0	122.4	5.385
TEC	26.4	76.4	5.428	41.1	127.0	5.258
PEG 300	11.9	34.6	6.630	23.6	69.0	6.221
MAN	52.7	121.8	2.785	64.0	181.8	4.598
SOR	37.9	104.4	5.164	50.1	160.0	5.137

## Data Availability

The data presented in this study are available in this article and Supplementary Materials.

## Supplementary Materials

The following supporting information can be downloaded at: https://www.mdpi.com/article/10.3390/pharmaceutics16040472/s1, Figure S1: Screw configuration. Image created with the ScrewConfig software (Version 1.42(2) Thermo Fisher Scientific Inc., Waltham, MA, USA); Figure S2: Scree-diagram left: PVA 3-82, right: PVA 4-88.
