# Supplementary material for: Understanding the Interaction of Thermal, Rheological, and Mechanical Parameters Critical for the Processability of Polyvinyl Alcohol-Based Systems during Hot Melt Extrusion
Source: Pharmaceutics. 2024 Mar 28;16(4):472. doi: 10.3390/pharmaceutics16040472 (PMC11055164; doi:10.3390/pharmaceutics16040472)
Supplement: Supplementary file 1 [file pharmaceutics-16-00472-s001.zip › pharmaceutics-2908177-supplementary.pdf]

Supplementary data

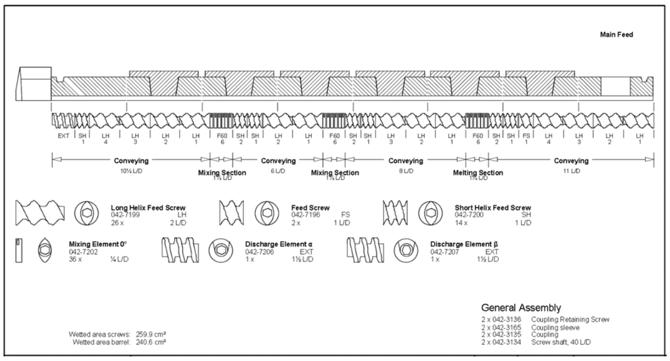

Figure S1. Screw configuration. Image created with the ScrewConfig software (Version 1.42(2) Thermo Fisher Scientific Inc., Waltham, MA, USA).

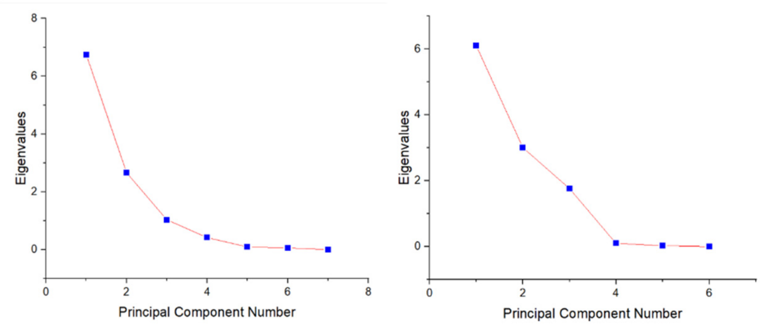

Figure S2. Scree-diagram (left): PVA 3-82, (right): PVA 4-88.
